# *In Vitro* Effects of Arthrocen, an Avocado/Soy Unsaponifiables Agent, on Inflammation and Global Gene Expression in Human Monocytes

**DOI:** 10.5539/ijc.v9n4p31

**Published:** 2017

**Authors:** Jared F Taylor, Ramin Goudarzi, Puya G Yazdi, Brian Allen Pedersen

**Affiliations:** 1 Systomic Health, USA; 2 Division of Research and Development, Pharmin USA, LLC, USA; 3 Department of Medicine, Division of Rheumatology, Allergy and Immunology, University of California, San Diego, USA

**Keywords:** monocytes, osteoarthritis, Arthrocen, avocado soy unsaponifiables, RNA-Sequencing, eicosanoid

## Abstract

Osteoarthritis (OA) is the most common form of arthritis. Symptomatically characterized by stiffness and pain, OA is a chronic degenerative disease of joints. Of note, there is growing interest in the potential use of plant-based compounds for symptomatic treatment of OA. Arthrocen is a plant-derived agent consisting of a one to two ratio of avocado and soy unsaponifiable extracts. In order to decipher the potential mechanisms of Arthrocen’s action at the molecular level, we employed an *in vitro* assay using cultured human THP-1 cells (a model cell line for monocytes) to study its effects. By pairing protein arrays enriched for inflammatory markers, transcriptomic pathway analysis using RNA-Sequencing, and eicosanoid specific lipidomics, we have begun to unravel its potential mechanism of action. Specifically, we found that Arthrocen can attenuate the inflammatory response at the transcript level while inducing significant changes in numerous cytokines. Furthermore, we discovered that while Arthrocen alone did not increase IL-8 or MCP-1 levels, its presence had a synergistic effect on the observed increase in response to LPS stimulation. Additionally, this synergistic effect of Arthrocen on LPS stimulation of IL-8 and MCP-1 protein levels was also observed at the mRNA level and suggests a regulatory mechanism at the transcriptional level. Interestingly, Arthrocen induced no changes in any of the eicosanoids studied. This multi-omics approach implies that Arthrocen functions at the level of gene transcription to dampen inflammation mediated by monocytes in OA.

## 1. Introduction

Osteoarthritis (OA) is the most prevalent form of arthritis. A chronic disease of joints, OA is symptomatically characterized by pain, stiffness and joint dysfunction. It is currently believed that mechanical stress plays a primary role in OA. Not surprisingly, high use joints, such as fingers, knees and hips are the most commonly affected joints. OA is a slow, progressive disease that is accompanied by articular cartilage damage, remodeling of the bone, new bone formation, and synovial inflammation and fibrosis (Malemud, 2015). An important identifying factor of OA is the breakdown of articular cartilage with underlying remodeling of the bone. While there is much debate about the significance of joint inflammation contributing to the pathology of OA, multiple studies have shown mononuclear cells in the synovial membrane in humans and there is additional evidence to support innate immune system activation in disease progression (Orlowsky & Kraus, 2015; Scanzello & Goldring, 2012). These findings give credence to the hypothesis that the presence of inflammation can increase breakdown of joint tissue while contributing to episodes of disease that are more painful and hence more symptomatic. OA patients suffer from impaired mobility and arthralgias. Palliative measures are the mainstay of current therapy with the goal of slowing disease progression and providing pain relief before joint replacement is ultimately indicated.

As most therapies primarily attempt to palliate symptoms, the use of plant-derived extracts in OA management is a therapeutic avenue with increased interest. One such avenue is the use of avocado/soy unsaponifiables (ASU) which comprise of unsaponifiable lipids extracted from the oil of both soybeans and avocados. The predominant composition of these unsaponifiable lipids are beta-sitosterol, campesterol, and stigmasterol, collectively referred to as phytosterols (Christiansen, Bhatti, Goudarzi, & Emami, 2015). The hypocholesterolemic effect of phytosterols is well characterized and likely mediated through their ability to impair cholesterol absorption and disrupt its endogenous biosynthesis (Smet, Mensink, & Plat, 2012). Additionally, further studies have shown the anti-inflammatory, antioxidant, and analgesic properties of phytosterols (Au, Al-Talib, Au, Phan, & Frondoza, 2007a; Bouic, 2001; de Jong, Plat, & Mensink, 2003; Gupta et al., 1980). Finally, recent clinical studies have also postulated ASU as having pain relieving and disease modifying effects in patients with OA (Appelboom, Schuermans, Verbruggen, Henrotin, & Reginster, 2001; Głuszko & Stasiek, 2016).

Arthrocen, an ASU containing agent, is comprised of 100 mg of unsaponifiable avocado extracts and 200 mg of unsaponifiable soybean extracts. These ASU extracts are sold in bulk under the trade name AvoVida. Prior analyses of Arthrocen by mass spectroscopy have shown that its major sterol component is comprised of dihydrocholesterol, campesterol, stigmasterol and β-sitosterol (Christiansen et al., 2015). Additionally, these particular sterols are in greater relative abundance in comparison to ASU extracts from other formulations (Christiansen et al., 2015). In order to decipher a potential mechanism of action, we designed an *in vitro* model by utilizing human THP-1 cells. THP-1 cells are a monocytic cell line derived from a patient with monocytic leukemia and have been extensively used to study monocyte and macrophage function. As peripheral blood mononuclear cells, and specifically monocytes, are thought to play an important role in the pathophysiology of OA, THP-1 cells can be used as a tool to investigate the therapeutic effects of substances on OA progression. Specifically, our goal was to test Arthrocen’s effects at therapeutic equivalent doses with a ‘multi-omics’ approach, which targeted genome wide transcription, immune response-related protein levels, and eicosanoids in human THP-1 cells.

## 2. Materials and Methods

THP-1 cells were incubated with Arthrocen or without (control media) in triplicate at a previously determined therapeutically equivalent concentration (25ug/ml) (Au, Al-Talib, Au, Phan, & Frondoza, 2007b). Of note, Arthrocen is manufactured as per the ASU product in Au, Al-Talib, Au, Phan, & Frondoza, 2007b in facilities inspected by the United States of America Food and Drug Administration under current Good Manufacturing Practices (GMP). Control media refers to the growth media used to culture THP-1 cells. LPS was used as a pro-inflammatory stimulus. All experiments consisted of the following four groups: THP-1 cells with control media, THP-1 cells with Arthrocen, THP-1 cells with control media stimulated with LPS, and THP-1 cells with Arthrocen stimulated with LPS. For each replicate within a given triplicate, THP-1 cells were harvested and its corresponding culture supernatant were collected for downstream analyses.

### 2.1 THP-1 Cell Culture and Avocado Soy Unsaponifiable Preparation

The THP-1 cell line (human monocyte; American Type Culture Collection (ATCC), Manassas, VA, USA) was cultured exactly as recommended by ATCC. RPMI-1640 medium and all supplements were manufactured by Gibco, Grand Island, NY, USA. Arthrocen (Pharmin USA, LLC, San Jose, CA, USA; avocado/soy unsaponifiables at a 1:2 ratio as per dry weight) was dissolved in 100% ethanol with continuous mixing at 50°C for 60 minutes. For 72 hours prior to the stimulation with LPS, 5 × 10^5^ THP-1cells/well of a six-well plate were incubated in cell media at 37°C, 5% CO2 for 72 hours with either vehicle (ethanol) or Arthrocen (25ug/ml) dissolved in vehicle (ethanol). After this pretreatment, cells were stimulated with LPS (20ng/mL; Sigma-Aldrich, St. Louis, MO, USA) for 6 hours. Following the 6-hour stimulation with LPS, culture supernatants for each replicate were aspirated and snap frozen in liquid nitrogen. THP-1 cells were then detached from the wells by the addition of 1mL of ice-cold PBS and subsequent scraping. This mixture of THP-1 cells in ice-cold PBS was then collected and centrifuged at 400 × g for 3 minutes at 4°C. The resulting supernatant was then aspirated for removal and the THP-1 cell pellets were snap frozen in liquid nitrogen.

### 2.2 Analysis of Inflammatory Factors in Culture Supernatants

Concentrations of 40 cytokines/chemokines within culture supernatants were determined in triplicate using the RayBiotech (Norcross, GA, USA) Quantibody® Human Inflammation Array 3 Kit (see Supplemental Table 1 for the list of cytokines/chemokines). Samples were supplied to and then assayed by RayBiotech. Briefly, array chambers were blocked and then 100ul of undiluted samples were incubated in the array chambers. Chambers were then washed five times and then incubated with the cocktail of biotinylated detection antibodies. The chambers were then washed five times and incubated with Cy3 equivalent dye-conjugated avidin. This was followed by an additional five washes. The fluorescent signal on the slides was then measured with an InnopsysInnoScan 710 (Chicago, IL, USA) at a wavelength of 532nm. The average of individual sample fluorescence in quadruplicate was used to determine actual protein concentrations (pg/mL) that were determined via standards incorporated into the array.

### 2.3 Eicosanoid Analysis

The comprehensive eicosanoid panel analysis was performed in triplicate on snap frozen cell pellets at the LIPID MAPS® Lipidomics Core at the University of California, San Diego. The methodology was as previously described (Currais et al., 2014).

### 2.4 RNA Extraction and RNA-Sequencing (RNA-Seq)

Total RNA was extracted in triplicate per experimental group from the snap frozen THP-1 cell pellets using Trizol (ThermoFisher Scientific, Carlsbad, CA, USA) as per the manufacturer’s protocol. The samples were then quantified using Nanodrop (ThermoFisher Scientific, Waltham, MA USA). Samples were then supplied to Applied Biological Materials, Inc. (Richmond, BC, Canada) for poly(A) enrichment and subsequent next-generation sequencing (~8 million reads per sample; 1x75bp single end). For each replicate within a triplicate, the FASTQ files were processed with TopHat (version 2.1.0) and reads aligned the to the reference human genome (GRCh38). Cufflinks (version 2.2.1) with default parameters and as per ENCODE (Encyclopedia of DNA Elements) Consortium guidelines was used to determine expression of individual transcripts(Trapnell et al., 2012; Trapnell, Pachter, & Salzberg, 2009). Prior to merging biological replicates for the determination of differentially expressed transcripts between experimental groups, their variability was assessed using the cuffdiff and cuffnorm tools within Cufflinks, respectively (See Supplemental Table 2 [Differentially expressed mRNA transcripts as per cuffdiff analyses of RNA-Sequencing data]).

### 2.5 Statistical Analysis

If a statistically significant difference was identified between groups with respect to the cytokines/chemokines assessed in the protein array or the eicosanoid panel as per one-way analysis of variance (ANOVA), the exact *p*-values were calculated as per *post-hoc* Tukey tests. Differentially expressed mRNA transcripts were identified as per the pipeline described above using the Cufflinks package with default settings. DAVID 6.7 was utilized to determine which biological processes were differentially regulated as per gene ontology (GO) clustering (Huang, Sherman, & Lempicki, 2009). Differentially regulated biological processes identified with GO clustering with a *p*-value threshold of < 10^−6^ were visualized using GOrilla (Eden, Navon, Steinfeld, Lipson, & Yakhini, 2009).

## 3. Results

**Figure 1:**
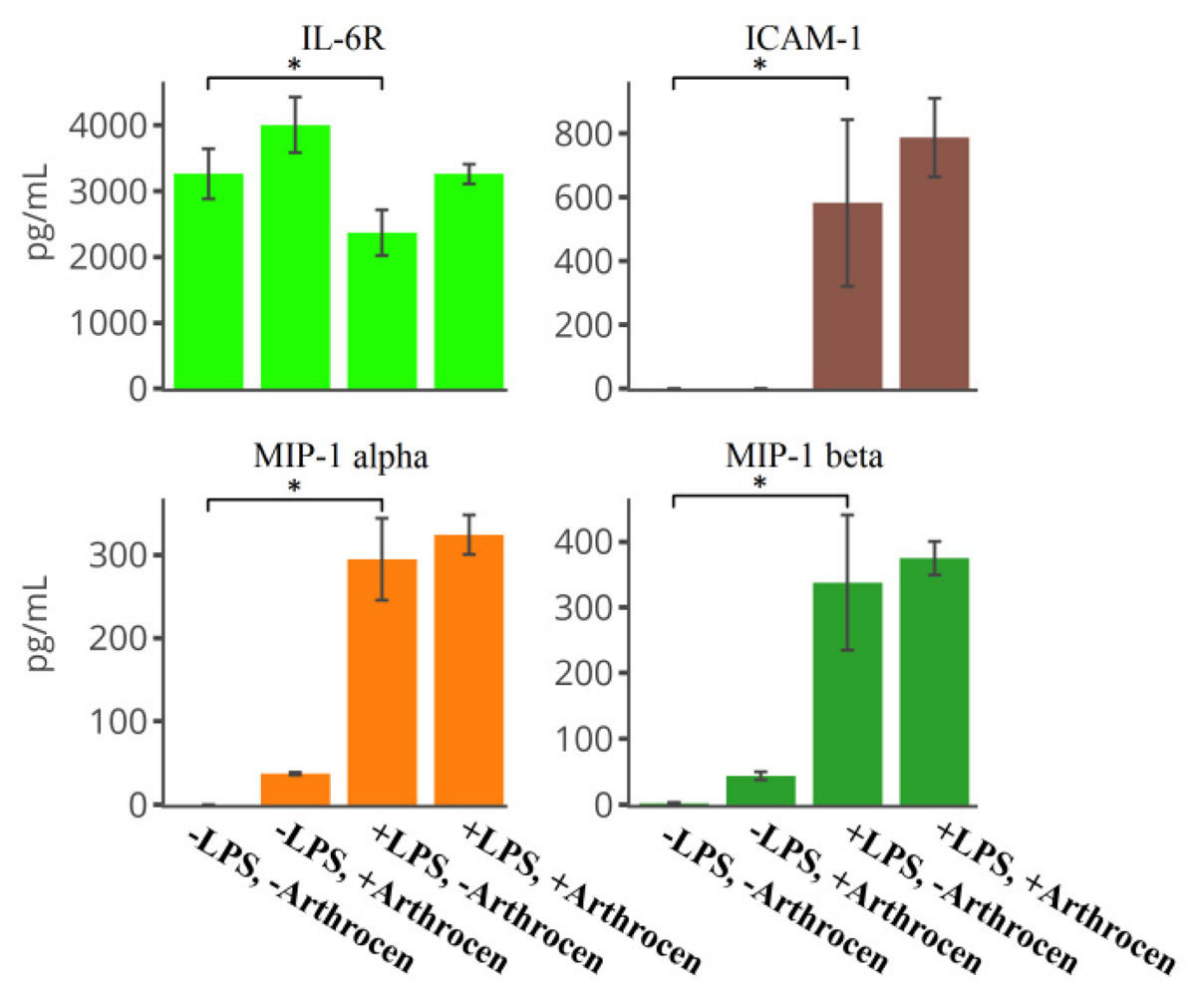
Pro-inflammatory cytokines independently increased by LPS as per ELISA: IL-6R, ICAM-1, MIP-1 alpha and MIP-1 beta. Overall effect, for a given cytokine is noted by a * between selected groups and representative of a *p*-value < 0.05 as per one-way ANOVA. Actual *p*-values as per *post-hoc* Tukey test are in Supplemental

**Figure 2:**
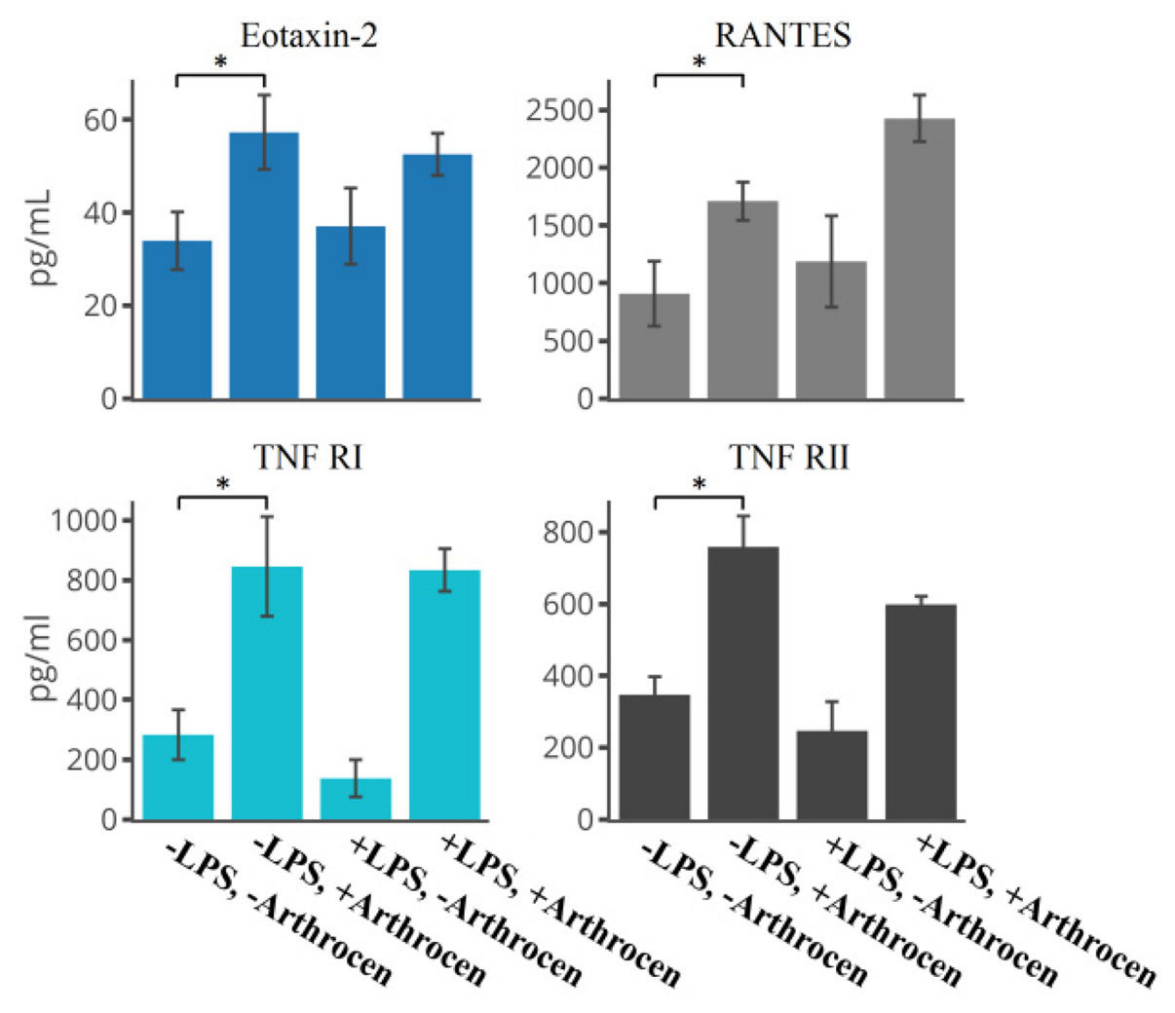
Pro-inflammatory cytokines independently increased by Arthrocen as per ELISA: Eotaxin-2, RANTES, TNF RI and TNF RII. Overall effect, for a given cytokine is noted by a * between selected groups and representative of a *p*-value < 0.05 as per one-way ANOVA. Actual *p*-values as per *post-hoc* Tukey test are in Supplemental Table 1.

**Figure 3:**
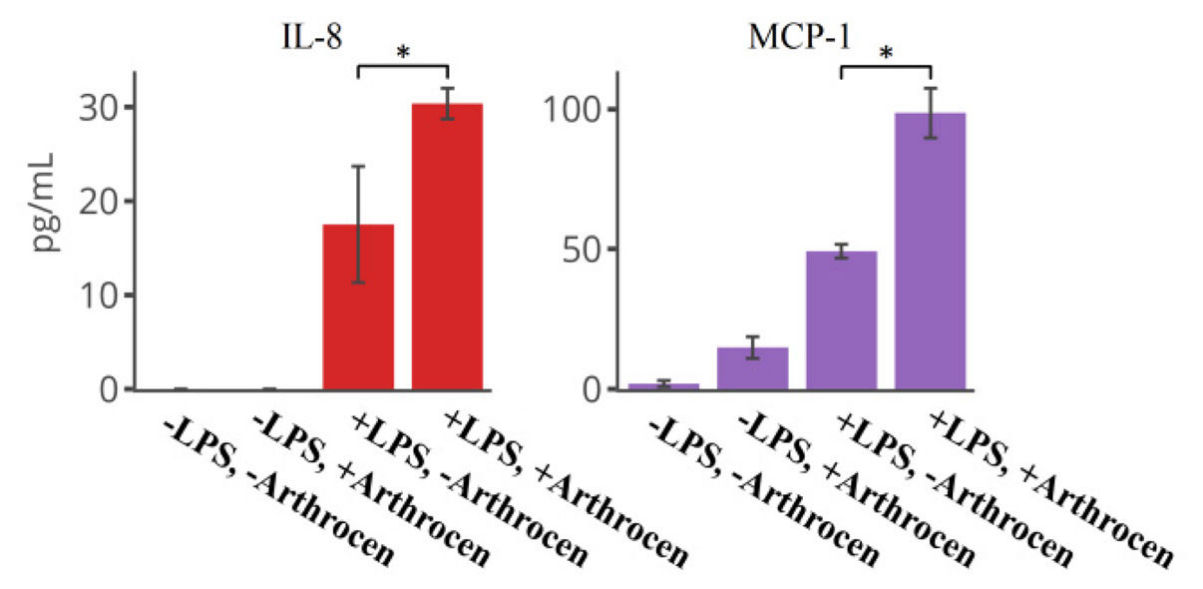
Pro-inflammatory cytokines independently increased by LPS with a synergistic increase in the presence of Arthrocen: IL-8 and MCP-1. Overall effect, for a given cytokine is noted by a * between selected groups and representative of a *p*-value < 0.05 as per one-way ANOVA. Actual *p*-values as per *post-hoc* Tukey test are in Supplemental Table 1

We utilized a protein array assay to screen secreted proteins from the human monocyte cell line (THP-1) in order to examine the effects of Arthrocen on inflammatory cytokines. We first incubated THP-1 cells with either media alone or in media supplemented with Arthrocen for 72 hours before an additional 6 hours of incubation with either vehicle alone or LPS. In total, we analyzed 40 proteins of which 13 were detectable (defined by its positive detection in all four replicates for a given experimental group; Supplemental Table 1). 12 out of these 13 proteins passed statistical significance when comparing differences between groups. LPS stimulation independently increased the levels of ICAM1, MIP-1 alpha, MIP-1 beta, IL-8, and MCP1 (Figures 1 and 3) while Arthrocen independently increased levels of Eotaxin-2, RANTES, TNF RI and TNF RII (Figure 2).

**Figure 4:**
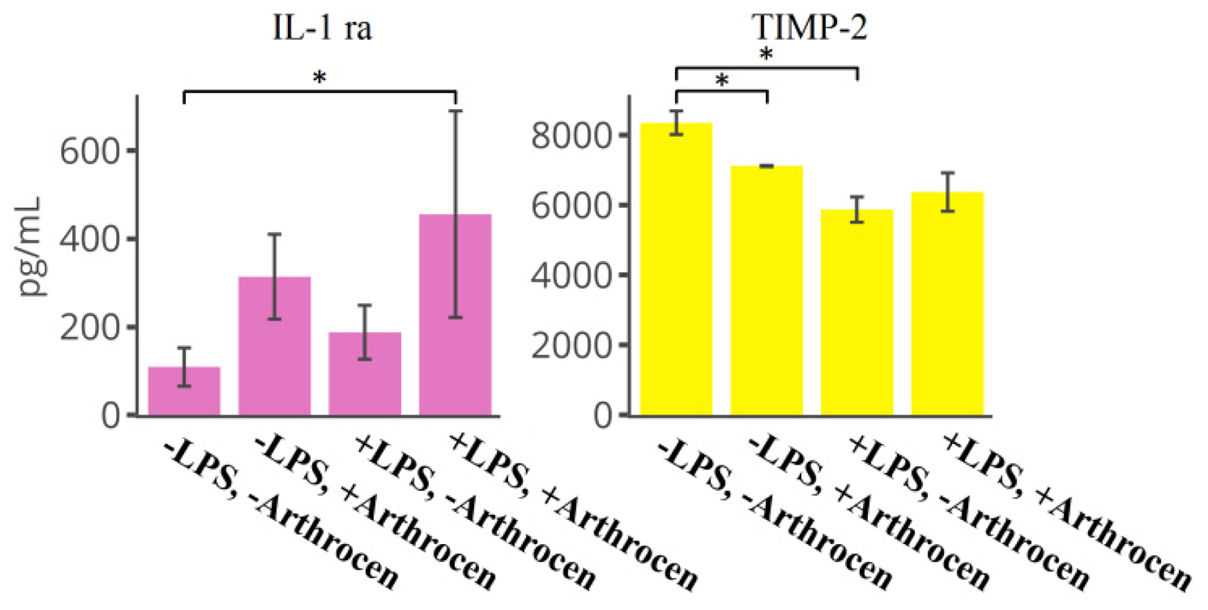
Pro-inflammatory cytokines significantly increased in the presence of both Arthrocen and LPS (IL-1 ra) or independent decreased by both Arthrocen and LPS (TIMP-2). Overall effect, for a given cytokine is noted by a * between selected groups and representative of a *p*-value < 0.05 as per one-way ANOVA. Actual *p*-values as per *post-hoc* Tukey test are in Supplemental Table 1

While incubation with Arthrocen alone did not increase IL-8 or MCP-1 levels, its presence had a synergistic effect on the observed increase in response to LPS stimulation (Figure 3). Additionally, LPS stimulation was associated with decreased levels of IL-6R and TIMP-2 (Figures 1 and 4). Arthrocen also independently decreased the levels of TIMP-2 but no combined effect with LPS was observed (Figure 4). LPS and Arthrocen had no individual effect on IL-1 ra, but in combination increased IL-1 ra levels (Figure 4). These results were indicative of an independent effect of Arthrocen on inflammatory cytokines, as well as, a modulatory effect of Arthrocen on the proinflammatory changes induced by LPS on a monocytic cell line.

**Figure 5:**
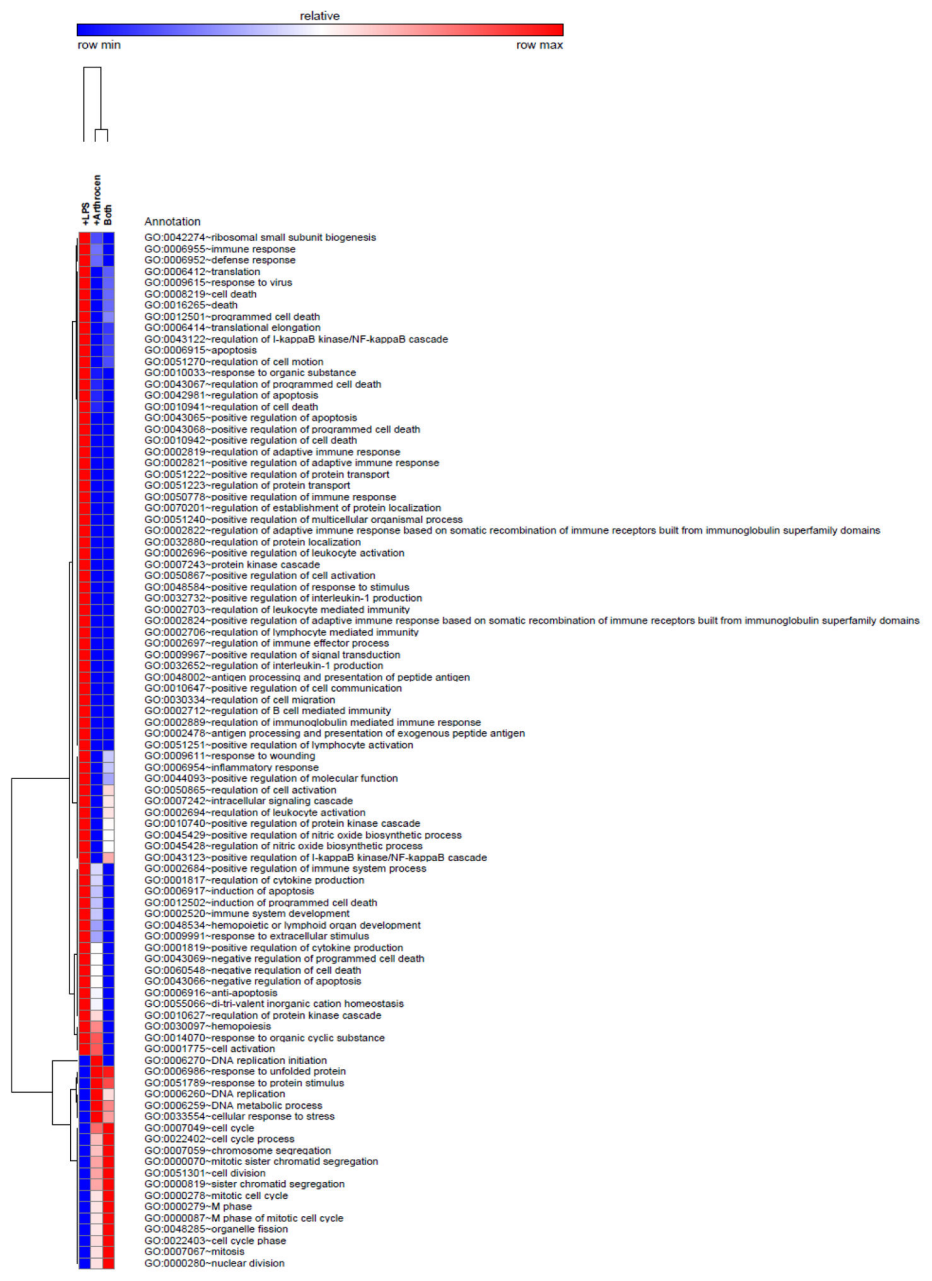
Graphical display of the effects of Arthrocen on gene expression as per clustering analyses of RNA-Sequencing data. Hierarchical clustering of GO clusters for biological processes with a statistically significant difference (q-value < 0.05) in a least one of the indicated comparisons

As the protein array data was indicative that Arthrocen can alter an inflammatory response, we continued to investigate Arthrocen’s effects by performing RNA-Seq of poly(A) enriched mRNA from the same samples used in the protein array to tease out its effects on global gene expression. We then performed computational GO analyses on the RNA-Seq data in order to highlight the biological processes that are potentially affected by Arthrocen. As the data demonstrates, hierarchical clustering of the GO terms (Figure 5) reveals that preincubation with Arthrocen is associated with a gene expression profile that is relatively stable upon acute stimulation with LPS. The majority of the similarly regulated gene clusters in comparison to the controls are related to the cell cycle or functions of the immune system that are characteristic of monocytes.

We proceeded to perform a lipidomic analysis utilizing an eicosanoid panel as these molecules are synthesized by monocytes and can act as messengers regulating pain pathways (Blotman, Maheu, Wulwik, Caspard, & Lopez, 1997). Of clinical relevance is the inhibition of the synthesis of some eicosanoids by nonsteroidal anti-inflammatory drugs (NSAIDs), a common class of pain medication used to treat the symptoms of OA. As some human studies on OA have demonstrated that ASU containing supplements decreases the usage of NSAIDs, it is biologically plausible that avocado/soy unsaponifiables have an effect on eicosanoid synthesis and levels (Appelboom et al., 2001; Głuszko & Stasiek, 2016; Kennedy, Stobo, & Goldyne, 1980). Samples were analyzed for a panel of 132 eicosanoids. In total we detected 7 different eicosanoid in the monocytes (Supplemental Table 3). However, no significant differences were detected between treatment groups.

## 4. Discussion

Since monocytes are believed to play a vital role osteoarthritis pathogenesis, we utilized THP-1 cells to determine Arthrocen’s effects on monocytes. Overall, our studies attempted to determine Arthrocen’s effects on both the basal state in addition to its ability to modulate the cellular response to an inflammatory trigger. We investigated these effects by quantifying protein levels of known inflammatory proteins, utilizing RNA-Seq to globally profile changes in gene expression levels at the mRNA level, and finally lipidomic profiling of eicosanoids since they function as mediators of cell signaling pathways involved in inflammation and pain. Our three-prong, unbiased analysis was utilized in order to better elucidate the potential effects of Arthrocen at the global level in mediating an immune response in addition to further defining which specific molecules it is directly influencing.

As can be seen by Figures 1 through 4, Arthrocen demonstrated a statistically significant effect on multiple cytokines. Interestingly, this effect was either independent of the pro-inflammatory response to LPS stimulation or synergistically increased with it. We did not find any attenuated LPS response in the presence of Arthrocen among the inflammatory markers we studied. While incubation with Arthrocen alone did not increase IL-8 or MCP-1 levels, its presence had a synergistic effect on the observed increase in response to LPS stimulation. Additionally, this synergistic effect of Arthrocen on LPS stimulation of IL-8 and MCP-1 protein levels was also observed at the mRNA level and suggests a regulatory mechanism at the transcriptional level (Supplemental Table 2). These findings demonstrate that Arthrocen clearly has an effect on inflammatory markers and cytokines within monocytes though the exact mechanism by which these effects can alter the progression or inflammation associated with OA is not clear. However, MCP-1 and IL-8 have both been detected in synovial fluid of joints affected by osteoarthritis, are well-described chemoattractants for leukocytes, and thought to contribute to both chondrocyte differentiation and collagen matrix degradation. This should be heeded when considering clinical usage of ASU for OA.

We next performed unbiased RNA-Seq to quantify changes at the messenger RNA level. As demonstrated in Figure 5, Arthrocen supplementation resulted in widespread and statistically significant changes to numerous transcripts involved in diverse pathways linked to inflammation. Of note, its addition resulted in attenuation of the effects of the pro-inflammatory agent LPS. Once again, this signifies that Arthrocen works at the gene level to attenuate inflammation in OA in monocytes. We next performed pathway analysis to further study these effects. As seen in Supplemental Figure 1, pathways involved in the formation of an immune response and chemotaxis were differentially regulated.

When comparing changes in protein levels as per the array with changes in transcriptional levels as per the RNA-Seq data, not all changes were concordant. This highlights the utility of a ‘multi-omics’ approach to further elucidate the mechanism behind complex biological responses.

We next performed lipidomic analysis of eicosanoid. Derived by the oxidation of 20-carbon fatty acids, eicosanoid are signaling molecules that regulate and induce changes over a wide array of both physiological and cellular systems: cellular growth, inflammation, immunity to toxic compounds and pathogens, and as CNS secondary messengers (De Caterina & Basta, 2001). Hence, some are classified as hormones. Interestingly, Arthrocen induced no changes in the levels of any of the eicosanoids studied. This raises the possibility that the affects observed with Arthrocen were not mediated through eicosanoids. However, prior studies have demonstrated that monocytes perhaps utilize arachidonic acid derived from other cell types for the synthesis of eicosanoids (Goldyne & Stobo, 1982; Petri, Thul, Ovchinnikova, & Bäck, 2015). To assess for the potential of our cells to make prostaglandins and leukotrienes (products downstream of arachidonic acid in eicosanoid biosynthesis) we assessed our RNA-Sequencing data for the transcripts involved in these pathways. The transcripts for the enzymes at the major branch points of eicosanoid production from arachidonic acid were present (PTGS1, PTGS2, ALOX5AP, ALOX5). PTGS1 and PTGS2 were not differentially regulated for any of the comparisons. However, the levels of ALOX5AP and ALOX5 were both statistically significantly increased with LPS stimulation (~1.67x and ~1.84x, respectively). In comparison to the group stimulated with LPS, ALOX5 transcripts levels were decreased ~40% in the group pre-incubated with ASU and then stimulated with LPS (see Supplemental Table 2). As such, a potential effect on these mediators of inflammation was perhaps not detected due to technical reasons as our system was of a single cell type and not supplemented with precursors of eicosanoids. Of note, our recent study on the effects of ASU on human chondrocytes suggest that this cell type is, at least in part, contributory to the production of eicosanoids involved in the pathogenesis of OA (Goudarzi, Taylor, Yazdi, & Pedersen, 2017).

Overall, our three-pronged approach revealed the underlying theme that Arthrocen attenuates the effects of numerous transcripts and pathways affected by LPS stimulation but without a corresponding attenuation in cytokine levels or eicosanoids. Taken together, these findings imply that the major effects of Arthrocen on monocytes are mediated through changes not detected by the measurement of classic cytokines and chemokines that have traditionally been used as biomarkers. Notwithstanding, a limitation of this study is that all data is derived from a tumor cell line in triplicate only. Additionally, we recognize that our descriptive study does not recapitulate the complex environment at the synovium or within the synovial fluid of a joint affected by osteoarthritis.

In conclusion, by utilizing an *in vitro* model of the effects of Arthrocen on monocytic cells, we were able to demonstrate that Arthrocen can play a significant role in attenuating inflammatory responses at the cellular level. Next steps would involve testing Arthrocen *in vitro* on mixed cell populations, *in vivo* in animal models of OA, and at the clinical level to determine its efficacy in a patient population.

## Supplementary Material

Figure 1

Table 1

Table 2

Table 3

## Abbreviations

ASU: avocado/soy unsaponifiable
GO: gene ontology
NSAIDs: nonsteroidal anti-inflammatory drugs
OA: osteoarthritis
RNA-Seq: RNA-Sequencing
