# Supplementary figures and images for: In Vitro Effects of Arthrocen, an Avocado/Soy Unsaponifiables Agent, on Inflammation and Global Gene Expression in Human Monocytes
Source: Int J Chem. Author manuscript; Available in PMC 2018 Apr 17. (PMC5903287; doi:10.5539/ijc.v9n4p31)

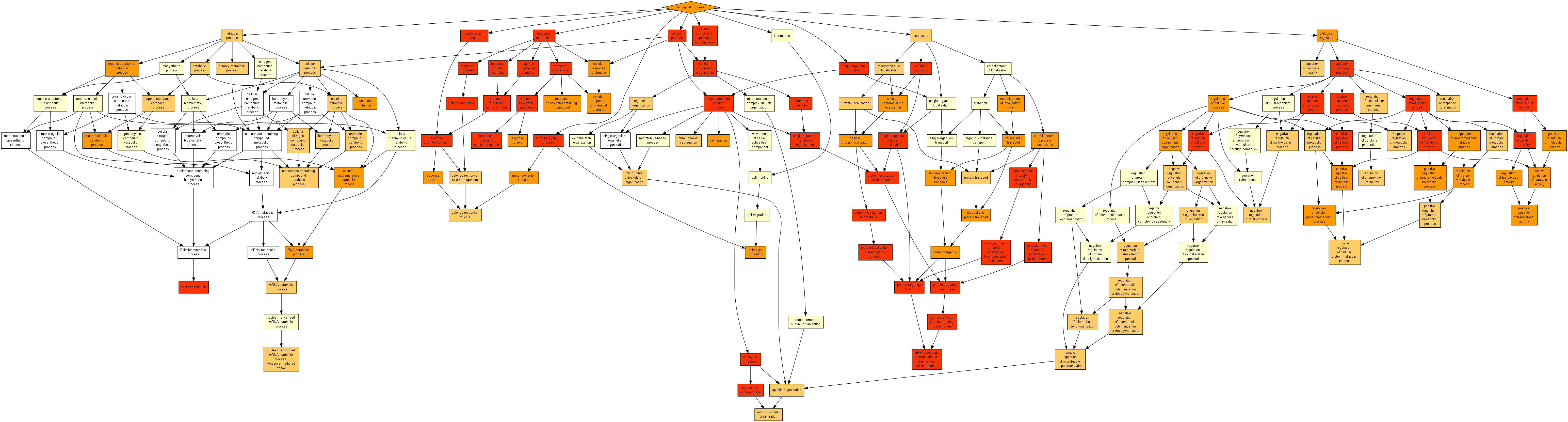

Supplement: Figure 1 [file NIHMS956886-supplement-Figure_1.png]
